# Imperial trophy or island relict? A new extinction paradigm for Père David's deer: a Chinese conservation icon

**DOI:** 10.1098/rsos.171096

**Published:** 2017-10-25

**Authors:** Samuel T. Turvey, Ian Barnes, Melissa Marr, Selina Brace

**Affiliations:** 1 Institute of Zoology, Zoological Society of London, Regent's Park, London NW1 4RY, UK; 2 Earth Sciences Department, Natural History Museum, Cromwell Road, London SW7 5BD, UK; 3 School of Geography, Royal Holloway University of London, Egham TW20 0EX, UK

**Keywords:** ancient DNA, conservation biology, *Elaphurus davidianus*, extinction dynamics, mammal extinction, range collapse

## Abstract

Determining the ‘dynamic biogeography’ of range collapse in threatened species is essential for effective conservation, but reconstruction of spatio-temporal patterns of population vulnerability and resilience can require use of non-standard ecological data such as historical archives. Père David's deer or milu, one of the few living mammal species that has become extinct in the wild, is historically known only from a small captive herd of unknown provenance that survived until 1900 in the Imperial Hunting Park near Beijing, from which all living individuals are descended. Using ancient DNA analysis, we demonstrate that two fawns collected in 1868 from Hainan Island, off the southern Chinese mainland, represent the only known wild milu specimens and were sampled from probably the last wild population. The Hainan milu population shows extremely low genetic differentiation from descendants of the Beijing herd, suggesting that this now-extinct population may have been the source of the captive herd. This revised extinction model refutes the supposed long-term survival of a captive milu herd for centuries or millennia after final extinction of wild populations, highlighting the vulnerability of remnant mammal populations in the absence of proactive management and the importance of historical museum collections for providing unique new insights on evolution, biogeography and conservation. Milu experienced a pattern of final population persistence on an island at the periphery of their former range, consistent with the ‘range eclipse’ or ‘contagion’ model of range collapse, and matching the spatial extinction dynamics of other extinct mammals such as the thylacine and woolly mammoth.

## 1. Introduction

Understanding the spatio-temporal pattern of population decline and persistence in threatened species is a key step to identify environmental conditions associated with vulnerability or resilience to anthropogenic threat factors, and therefore to inform effective conservation [1–4]. However, extinction events may take decades, centuries or longer to run their course, and so developing a robust evidence-base on the ‘dynamic biogeography’ of range collapse may necessitate incorporating novel data from long-term historical archives as well as recent ecosystems, to avoid incomplete or biased reconstruction of past distributions and ecological requirements for threatened species [5,6]. This approach is particularly important for species reduced to tiny remnant populations or that are already extinct in the wild, which have current-day geographical distributions that lack a true evolutionary or ecological basis due to disruption by human activity.

**Figure 1.**
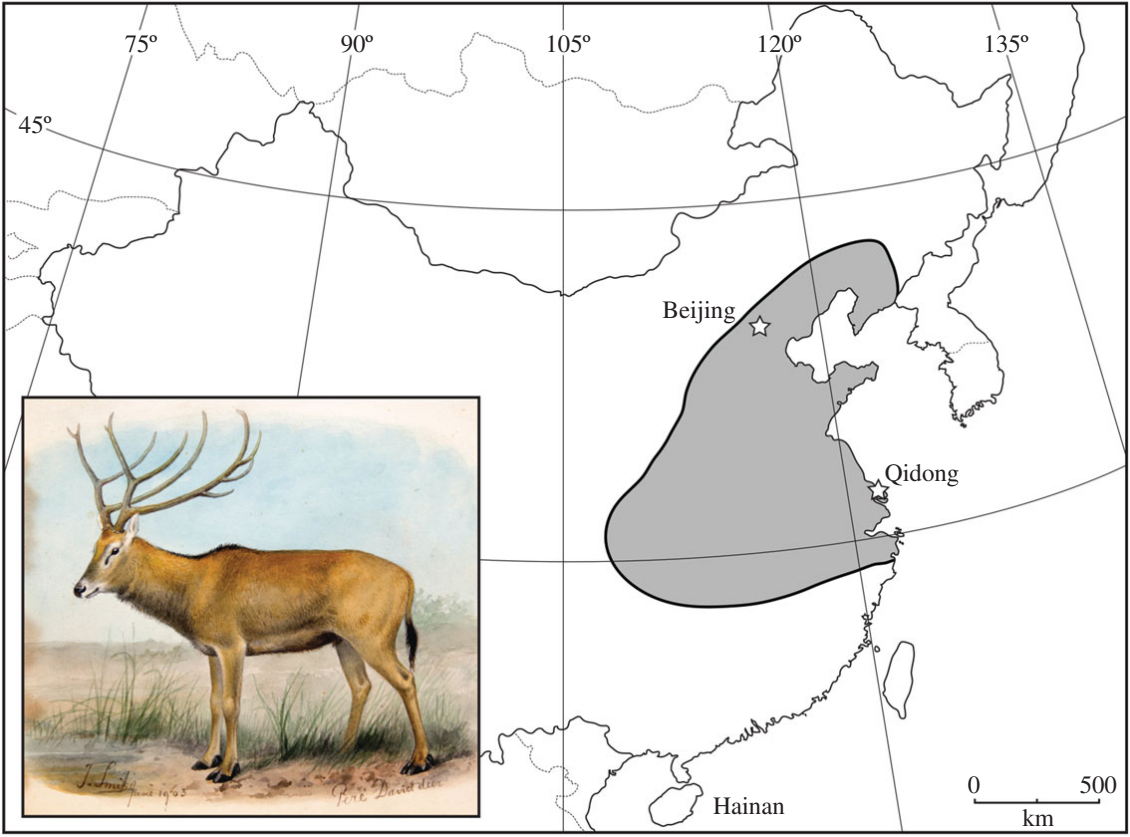
Map of China, showing locations of Beijing, Qidong and Hainan, and the inferred Holocene range of milu from Ohtaishi & Gao [7] (grey). Inset, previously unpublished painting of a milu from 1903 in the library of the Zoological Society of London.

Père David's deer or milu (*Elaphurus davidianus*) is one of the few living mammal species that has become totally extinct in the wild in recent centuries (figure 1). The story of its near-extinction and subsequent recovery is famous. A captive herd of deer unknown to Western science was discovered in 1865 by the French missionary Père Armand David at the Imperial Hunting Park at Nanhaizi, outside Beijing in northern China; individuals were sent to several European collections, but the original herd was then wiped out by hunting following flooding in 1894 and the Boxer Rebellion in 1900 [8]. The last 18 European individuals, of which only 11 were capable of reproducing, were gathered by the 11th Duke of Bedford at Woburn Abbey in the early twentieth century [8,9]. The first captive animals were returned to China in 1956, and the Chinese population now stands at over 2000 individuals [10].

The provenance and history of the Beijing herd are unknown. Milu are abundant in the Holocene record of northern and eastern China as far north as Beijing, but were apparently extirpated from this region long before Père David's discovery of the species at Nanhaizi [11]. The most recent possible milu record from mainland China dates from the reign of the Jiaqing Emperor (1796–1820), with the last reported wild population located approximately 1100 km southeast of Beijing, in Qidong County, Jiangsu Province [12] (figure 1). Milu were culturally important animals in ancient China [11,13,14] and were kept in imperial parks as high-status animals and sources of royal venison from at least the fifth century BCE [11,15]. The prevailing conservation model assumes the Beijing herd originated from a local mainland source population and may have been artificially managed in isolation at Nanhaizi for centuries or even millennia following the decline of milu populations across the mainland [8,9,11,16].

**Figure 2.**
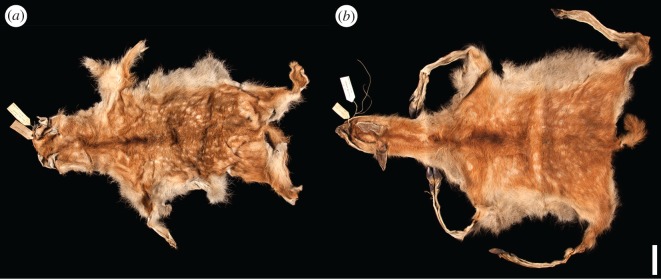
Skins of two wild-caught milu fawns collected on Hainan by Robert Swinhoe in 1868, representing the only known historical wild milu specimens. (*a*) NHM 70.2.10.28 and (*b*) NHM 70.2.10.29. Scale bar, 10 cm.

Hainan, China's southernmost province, is a 34 000 km^2^ island with a subtropical–tropical climate in the South China Sea, situated approximately 2300 km southwest of Beijing (figure 1). Today, Hainan contains wild populations of three deer species (Eld's deer *Panolia eldii*; sambar *Rusa unicolor*; red muntjac *Muntiacus vaginalis*) [17], and the island's limited fossil and zooarchaeological records lack evidence of milu [18,19]. However, a report of possible wild milu on Hainan was made in 1904, but was largely discounted later that year [20,21]. The skins of two wild-caught fawns collected on Hainan by Robert Swinhoe in 1868 and currently accessioned in the Natural History Museum (NHM), London (NHM 70.2.10.28, 70.2.10.29), which were originally identified as Eld's deer [22], have also subsequently been suggested to possibly represent milu based on hair whorl patterns [23] (figure 2). These suggested reports have been generally ignored by subsequent researchers [7,9,10,16,17]. In order to reconstruct milu population history and the evolutionary affinities of Hainan's mammal fauna, we conducted ancient DNA analysis of Swinhoe's mystery deer specimens and reveal an unexpected new model for the dynamic biogeography of one of China's most iconic threatened mammals.

## 2. Material and methods

### 2.1. Laboratory

DNA extractions and library preparations were conducted in a dedicated ancient DNA laboratory (NHM, London). DNA was extracted from NHM 70.2.10.28 and NHM 70.2.10.29 using a QIAamp DNA Micro kit and protocols, with the inclusion of a second final elution step. Library preparations followed a modified version of the protocol in [24], with modifications as follows: the initial DNA fragmentation step was not required; and all clean-up steps used MinElute PCR purification kits (Qiagen). The index PCR step used AmpliTaq Gold DNA polymerase and the addition of 0.4 mg ml^−1^ BSA. The index PCR was set for 20 cycles with three PCRs conducted per library. The library was sequenced on an Illumina NextSeq platform (NHM, London) using a NextSeq 500/550 Mid-Output v2 Kit (75 bp paired end).

### 2.2. Bioinformatics

AdapterRemoval [25] was used to trim residual Illumina adapter sequences and low-quality bases, with paired-end reads longer than 25 bases merged with a minimum overlap of 11 bases. The quality-trimmed, merged-only reads were initially aligned to a reference mitochondrial genome of *P. eldii* (GenBank accession number: HM138200), and the consensus sequence was BLASTed against the NCBI Reference Sequence database. The reads were then realigned to a mitochondrial genome of a recent descendant of the Beijing milu herd (GenBank accession number: JN632632) using BWA [26], with a minimum base quality set to Phred scale 15. SAMtools (http://samtools.sourceforge.net) was used to further filter the mapped reads by map quality value 30 and to remove all duplicates.

### 2.3. Phylogenetics

Sequencing results from NHM 70.2.10.28 were comparatively poor (table 1), and so all further analyses were limited to NHM 70.2.10.29 only. The consensus sequence for the near-complete mitochondrial genome of NHM 70.2.10.29 (electronic supplementary material; GenBank accession number: MF673864) was aligned with modern Cervinae sequence data available on GenBank (electronic supplementary material, table 1). For the aligned mitochondrial genome dataset (D-loop removed), DNA substitution model and partition fit were selected under the Bayesian information criterion using PartitionFinder 1 [27]. Bayesian trees were constructed using MrBayes 3.2 [28], applying nucleotide substitution models identified as the best-fitting by PartitionFinder (electronic supplementary material, table 2), using four chains (three heated and one cold) run for 1 × 10^6^ generations and sampling every 1 × 10^3^ generations with a burn-in period of 250 trees. Nodal support was determined by approximate posterior probabilities performed in MrBayes. To further assess the level of sequence divergence within phylogenetically closely related deer species, we additionally aligned modern cytochrome *b* (cyt *b*) sequence data from GenBank (table 2) for samples of *E. davidianus* (from other recent descendants of the Beijing milu herd) and *P. eldii*, and calculated pairwise sequence divergence estimates using mega 5.1 [29] and the Kimura-2 parameter (K2P) model.

**Table 1.** Number of sequenced reads mapping to either *P. eldii* or *E. davidianus* mitochondrial genomes.

		reads mapped		quality reads (i.e. no duplicates)	
sample	total reads	*P. eldii*	*E. davidianus*	*P. eldii*	*E. davidianus*
NHM 70.2.10.28	5 033 347	475	623	353	588
NHM 70.2.10.29	5 607 552	1762	2395	1266	2190

**Table 2.** Pairwise distances matrix, showing the number of base pair differences across the whole cyt *b* gene between NHM 70.2.10.29 and specimens of *P. eldii* and *E. davidianus* available on GenBank.

specimen number	species and specimen number					mean (±)
	*Panolia eldii*					
	AY157735	FJ556560	DQ249813	EU878390	AY607037	
AY157735						
FJ556560	8					
DQ249813	12	10				
EU878390	8	0	10			
AY607037	18	14	22	14		11.6 ± 6.02
NHM 70.2.10.29	53	55	59	55	63	
	*Elaphurus davidianus*					
	AF423194	JN632632	NC_018 358	JN399997		
AF423194						
JN632632	1					
NC_018358	1	0				
JN399997	1	0	0			
NHM 70.2.10.29	4	3	3	3		3.25 ± 0.5

## 3. Results

DNA sequenced from NHM 70.2.10.28 and NHM 70.2.10.29 were initially aligned to a reference mitochondrial genome for *P. eldii*. However, BLAST results indicated that the highest identity match for the consensus sequence on the NCBI Reference Sequence database for both samples was *E. davidianus.* Subsequent realignment of the sequenced DNA (reads) to an *E. davidianus* mitochondrial genome produced a higher number of unique quality mapped reads for both samples (table 1).

**Figure 3.**
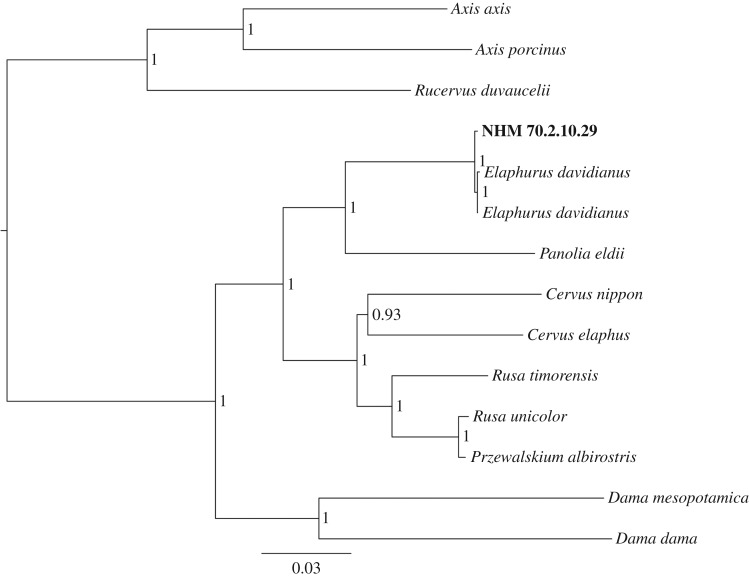
Bayesian phylogeny of cervines using near-complete* mitochondrial genomes and showing the phylogenetic placement of NHM 70.2.10.29. Numbers at nodes represent Bayesian posterior probabilities. Scale bar represents the number of substitutions per site. (*D-loop removed due to saturation of sites in this hypervariable region distorting the phylogenetic signal.)

Bayesian phylogenetic analyses comprising mitochondrial genomes for extant cervine species and NHM 70.2.10.29 produced a well-supported phylogeny identifying NHM 70.2.10.29 as *E. davidianus* (figure 3). Results from within-species cyt *b* sequence divergence analyses further confirm NHM 70.2.10.29 as being a milu as opposed to Eld's deer and illustrate the low levels of within-species sequence divergence between NHM 70.2.10.29 and recent descendants of the Beijing milu herd (table 2).

## 4. Discussion

Our ancient DNA analysis of approximately 150-year-old museum specimens demonstrates conclusively that Swinhoe's wild deer collection from Hainan includes milu fawns, with these skins representing the only historical wild milu specimens known anywhere in the world. This newly confirmed historical locality record is considerably outside the known Holocene range of the species in China (figure 1) and thus provides an important new example of ecological and biogeographical biases that can result from incomplete sampling across species' ranges and inaccurate reconstruction of historical baselines. Although Swinhoe is also known to have sent some of the first milu individuals, including fawns, from the Beijing herd to the UK in the 1860s [8], the provenance of his Hainan collection is well documented [22], precluding the possibility of accidental confusion of specimens. Hainan therefore appears to have been home to China's last known wild milu population, which was still extant when Père David first saw the captive herd at Nanhaizi. How long it survived beyond the 1860s is unclear. Swinhoe acquired his specimens from local hunters, reporting that the animals he identified as Eld's deer were ‘rarely brought from the mountains’ (likely referring generically to the interior of Hainan) and that he ‘had much difficulty in procuring the skins’ [22], suggesting that milu may have already been almost extinct. However, in light of our discovery of a definite nineteenth-century wild population, the dubious 1904 report of milu on Hainan [20,21] may indicate twentieth-century survival, and deer specifically named as ‘milu’ are recorded in Hainan's local *difangzhi* gazetteer record until 1917, although within a confusing list of 12 named deer ‘types’ lacking further description that cannot be assigned easily to known species [30].

The discovery of nineteenth-century or later survival of milu on Hainan reveals an alternative extinction model for this icon of Chinese conservation. Instead of persisting in captivity for centuries or millennia after final extinction of wild source populations, an apparently unique scenario before the recent development of carefully managed *ex situ* conservation programmes, the dynamic biogeography of milu decline shows a pattern of final population persistence at the southern margin of the species' known Holocene distribution which matches the ‘range eclipse’ or ‘contagion’ model of range collapse—where the spatial pattern of range contraction is determined by the directional spread of anthropogenic extinction factors, and populations typically survive longest in refugia along the edge of a historical range which are impacted last by these extinction forces [1–4]. The milu now joins a long list of other threatened or now-extinct species, also including thylacine (*Thylacinus cynocephalus*) and woolly mammoth (*Mammuthus primigenius*), which survived longest as remnant populations on islands at the periphery of their former ranges [1,2,4,31,32]. Northeastern China supported high human population densities during the Late Imperial period, and large mammals were likely to have been highly vulnerable to habitat loss and hunting associated with this demographic expansion [33,34]; conversely, Hainan, a marginal southern territory in China's Qing Dynasty empire, remained sparsely populated and economically undeveloped into the twentieth century [18]. A similar pattern of remnant population persistence for declining mammals in areas experiencing reduced human pressures at the southwestern margin of eastern Asia, following wide-scale extirpation across China during past centuries or millennia due to regional human population growth, has also recently been demonstrated using zooarchaeological and historical archives for several other species, including cervids [35,36].

Our genetic data also shed new light on the likely provenance and history of the captive Beijing milu herd. Our comparison of pairwise sequence divergence estimates for NHM 70.2.10.29 compared with recent descendants of the Beijing milu herd (0.26%, mean base pair differences = 3.25 ± 0.5), when matched against within-species differences seen across individuals sampled from allopatric Southeast Asian populations of the milu's extant sister species *P. eldii* (1.05%, mean base pair differences = 11.6 ± 6.02), indicates a much lower level of within-species variance for milu (table 2). Comparison of within-lineage divergence estimates available for other cervid species that have large geographical ranges, with populations occurring across continental areas comparable in extent to the distance between Hainan and northeastern China (red deer, *Cervus elaphus*: 1.4%; sika deer, *C. nippon*: 1.9%) [37], further highlights the limited variance seen between milu individuals sampled from the wild Hainan population and from the captive Beijing herd. Although the descendants of the Beijing herd have passed through a population bottleneck and exhibit low current genetic diversity [38,39], such recent demographic structuring would not be expected to affect estimates of the affinity of this herd to the extinct Hainan population. Indeed, cervids that have native populations distributed across eastern and southern China exhibit considerable population structuring and incomplete gene flow over this geographically heterogeneous region; the taxonomy of many of these allopatric populations remains debated, and most are interpreted as being differentiated at the subspecies or even species level [7,17,40]. Conversely, although levels of genetic diversity within Hainanese cervids have not yet been investigated, wild populations of other mammals show more limited genetic differentiation between landscapes across Hainan [41]. These comparisons therefore strongly suggest that historical and recent milu specimens are likely to have originated from a wild source population within a much more restricted geographical region.

We consider it likely that the late-surviving Hainan population may therefore have been the wild source population for the nineteenth-century Beijing herd, although we note that in the absence of genetic data from extinct mainland Chinese populations, we cannot rule out the possibility of generally low intraspecific variation across allopatric milu populations, so that the Hainan population might not be the only one that was phylogenetically close to the Beijing herd. There is evidence of ancient human transportation of other continental deer species to several island systems [42–45], raising the possibility that the Hainan milu population could itself have been originally introduced from mainland China. However, although this possibility cannot be discounted, ancient deer introductions were typically made to islands that lacked native large deer, and the local presence on Hainan of other surviving deer species that are considered native [17], together with the low cultural and economic importance of the island during most of China's history, makes it more likely that milu were native to Hainan.

As the Hainan population survived at least until the mid-nineteenth century, we consider it unlikely that the Beijing herd persisted in isolation for centuries or longer, and was instead more likely to have been stocked with individuals that had been translocated from Hainan much more recently. Although there is no direct documentary evidence of milu being sent as tributes from Hainan to Beijing, other economically and/or culturally important or otherwise unusual wild animals were sent regularly from Hainan to the mainland during this period [18,22,46]. As deer referred to as ‘milu’ are also recorded regularly in Hainanese Imperial-era *difangzhi* gazetteers [30], which functioned as hand-over documents for civil servants and include listings of significant local environmental products, the species may have been locally familiar as an important official resource. This revised history for the origin of the Beijing herd therefore challenges previous assumptions about milu ecology derived from this herd, such as hypotheses about the duration of time that antipredator behaviours may be maintained in captivity [16]. More fundamentally for conservation biology, this revised history refutes a supposed example of long-term survival of a small, isolated population of a large-bodied mammal species, thus highlighting the intrinsic vulnerability of such remnant populations in the absence of proactive conservation management (cf. [35]).

Asia's ecosystems are now experiencing extreme anthropogenic pressure and contain the world's highest levels of threatened biodiversity [47,48]. Developing an improved understanding of past environmental baselines and the history of faunal responses to regional human activities is therefore an urgent priority to predict likely future patterns of population survival or loss. Indeed, Hainan has experienced severe faunal depletion over the past century, which has escalated during recent decades [49–52], and one of its few endemic mammal species, the Hainan gibbon (*Nomascus hainanus*), is now probably the world's rarest mammal, with a global population of only 26 known individuals [41,53,54]. Recognition that the milu is probably native to Hainan and survived here later than elsewhere across its former range contributes to the growing body of knowledge on historical Chinese mammal extinctions [35,36] and provides important new insights for conservation of this threatened species and for environmental management of Hainan's natural landscapes—including the possibility of future reintroduction of descendants of the Beijing milu herd to their original distribution, rather than to more northerly regions of mainland China that may be ecologically unsuitable in the long term for a tropical-adapted population [10]. Our study also represents a new example of how historical museum collections can provide unique new insights into the evolution, ecology and biogeography of species that have experienced pre-modern range declines (cf. [55]), and emphasizes the value of underused archival resources for informing current-day conservation and preventing future extinctions in China's highly threatened mammal fauna.

## Supplementary Material

Table S1; Table S2

## Supplementary Material

Text-file S1

## Data accessibility

The data supporting this article are available as the electronic supplementary material.
