# Supplementary material for: Imperial trophy or island relict? A new extinction paradigm for Père David's deer: a Chinese conservation icon
Source: R Soc Open Sci. 2017 Oct 25;4(10):171096. doi: 10.1098/rsos.171096 (PMC5666285; doi:10.1098/rsos.171096)
Supplement: Table S1; Table S2 [file rsos171096supp1.docx]

**SI TABLES**

**Table S1.** Details of Genbank data used in our analyses.

| **Accession Number** | **Species** | **Mt DNA** |
| --- | --- | --- |
| JN399997 | *Elaphurus davidianus* | Whole mt genome |
| JN632632 | *Elaphurus davidianus* | Whole mt genome |
| HM138200 | *Panolia eldii* | Whole mt genome |
| HM049636 | *Przewalskium albirostris* | Whole mt genome |
| AB245427 | *Cervus elaphus* | Whole mt genome |
| AB210267 | *Cervus nippon* | Whole mt genome |
| JN632699 | *Rusa timorensis* | Whole mt genome |
| EF035448 | *Rusa unicolor* | Whole mt genome |
| JN632696 | *Rucervus duvaucelii* | Whole mt genome |
| NC_020680 | *Axis axis* | Whole mt genome |
| JN632600 | *Axis porcinus* | Whole mt genome |
| JN632629 | *Dama dama* | Whole mt genome |
| NC_024819 | *Dama mesopotamica* | Whole mt genome |
| AY157735 | *Panolia eldii* | Cytochrome *b* |
| FJ556560 | *Panolia eldii* | Cytochrome *b* |
| DQ249813 | *Panolia eldii* | Cytochrome *b* |
| EU878390 | *Panolia eldii* | Cytochrome *b* |
| AY607037 | *Panolia eldii* | Cytochrome *b* |
| AF423194 | *Elaphurus davidianus* | Cytochrome *b* |
| JN632632 | *Elaphurus davidianus* | Cytochrome *b* |
| NC_018358 | *Elaphurus davidianus* | Cytochrome *b* |
| JN399997 | *Elaphurus davidianus* | Cytochrome *b* |

**Table S2.** Partitions and models applied in phylogenetic analyses, as selected for by PartitionFinder.

| **Partition** | **Model** |
| --- | --- |
| 12s, 16s, ATP61, ATP81, ATP82, CYTB1, ND21, ND41, ND51, tRNA1, tRNA10, tRNA12, tRNA2, tRNA5, tRNA7, tRNA8, tRNA9 | GTR + G |
| ND62, ND63, tRNA11, tRNA3, tRNA4, tRNA6 | HKY + G |
| COX11, COX21, COX31, ND11, ND31, ND4L1 | SYM + G |
| COX12, COX22, COX32, CYTB2, ND12, ND32, ND42, ND4L2 | HKY + G |
| COX33, CYTB3, ND13, ND23, ND33, ND43, ND4L3, ND53, ND61 | GTR + G |
| ATP62, ND22, ND52 | HKY + G |
| ATP63, ATP83, COX13, COX23 | HKY + G |
